# Integration of Evidence Based Medicine into a Medical Curriculum

**DOI:** 10.3885/meo.2009.F0000225

**Published:** 2009-09-20

**Authors:** H M Tamim, M Ferwana, E Al Banyan, I Al Alwan, AH Hajeer

**Affiliations:** * College of Medicine, King Saud bin Abdualziz University for Health Sciences, Riyadh, Saudi Arabia; † King Abdullah International Medical Research Center, Riyadh, Saudi Arabia; ‡ Department of Family medicine, King Abdualziz Medical City, National Guard Health Affairs, Riyadh, Saudi Arabia; § Department of Pediatrics, King Abdualziz Medical City, National Guard Health Affairs, Riyadh, Saudi Arabia; ǁ Department of Pathology & Laboratory Medicine, King Abdualziz Medical City, National Guard Health Affairs, Riyadh, Saudi Arabia

**Keywords:** Evidence based medicine (EBM), medical education, medical curriculum, epidemiology

## Abstract

The College of Medicine at King Saud bin Abdulaziz University for Health Sciences (KSAU-HS) was established in January 2004. The four-year curriculum was based on the Problem Based Learning (PBL) format and involved the web-based graduate medical program adopted from the University of Sydney, Australia. At KSAU-HS, one additional semester was added to the beginning of this curriculum to prepare the students in English language skills, PBL, Information Technology and Evidence Based Medicine (EBM). EBM is part of the Personal and Professional Development (PPD) theme of the medical curriculum and is integrated into each stage of the medical curriculum. These modifications of the University of Sydney curriculum are presented here as a model of EBM integration into a college of medicine curriculum.

Evidence-based medicine (EBM) involves the use of current best evidence in making clinical decisions about an individual patient's care.^1^ EBM helps health care professionals overcome practice variation and cope with the rapidly increasing number of diagnostic and treatment options. In addition, EBM supports the movement toward patient participation in decision making.^2^

Adding EBM to medical curricula enables students to develop EBM skills from the outset of their clinical training and fosters an ongoing desire to practice using EBM.^3^ The main purpose of integration was to reinforce to the students EBM as a learning process, which is the theme of the adopted curriculum at the College of Medicine at King Saud bin Abdulaziz University for Health Sciences (KSAU-HS). Moreover, EBM is an expertise a health care provider must have to help in day-to-day decision making. Thus, integration of the EBM within the curriculum is important so as not to isolate it as a separate discipline.

The College of Medicine at KSAU-HS was established in January 2004. It is integrated within the King Abdulaziz Medical City in Riyadh, a multi-facility medical city including a 900-bed tertiary-care hospital that serves as the primary teaching center for the medical students. It is the first college of medicine in the Kingdom that requires a prior Baccalaureate degree rather than simply high school graduation for admission. Students applying to the College of Medicine at KSAU-HS must have a baccalaureate degree in Science, Allied Health Sciences, Pharmacology or Veterinary Medicine. The first cohort of thirty students was enrolled in September 2004.

**King Saud bin Abdulaziz University for Health Sciences College of Medicine** - The College of Medicine at KSAU-HS utilizes a four-year Problem Based Learning (PBL) web-based graduate medical program adopted from University of Sydney, Australia.^3^ To prepare the College for the new curriculum the Department of Medical Education was created along with several committees. The University of Sydney curriculum needed to be altered so that it was compatible with and reflected Saudi priority health problems and met the rules and regulations of the Kingdom of Saudi Arabia's Ministry of Higher Education.

Several working groups, including those for Student Assessment, Basic Clinical Sciences, Clinical Diagnostic, Clinical Communications, Procedure Skills, Community/Doctor, OSCE/OSPE and Evidence Based Medicine, reported to the Curriculum Committee. Each working group had specific charges to review the details of each block, to modify, change, or accept the initial curriculum presented by the University of Sydney, and to present their findings to the Curriculum Committee. EBM was part of the Personal and Professional Development Theme of the medical curriculum. We applied some changes to make EBM an integrated part of each stage of the four-year medical curriculum. In this paper we will highlight the new locally-developed EBM curriculum in the College of Medicine at KSAU-HS and show how we modified the original program to fit curricular needs.

Students enrolled in the medical program spend the first semester in a preparatory phase specifically developed by our faculty. During this semester (Phase I), students are acquainted with the different disciplines important for their initial medical studies. They are then enrolled in the Sydney program for phase II and III.^3^

Next we describe our modification for the pre-clinical years, phase I and II.

**Phase I** – This is an introductory semester of 16 weeks duration, including five blocks: English language skills and medical terminology, Introduction to PBL, Information Technology, Medical Ethics and Islamic Values, and finally, EBM and research methodology.

Table 1 shows the different topics introduced in the EBM and research methodology block. The objective of this block is to introduce students to the concept of EBM, including study designs, statistical analyses, literature search for the evidence, and basic research methodologies. This block focuses on experiential learning in terms of literature search and statistical analysis.

**Table 1.** Topics included in the EBM block included in Phase 1 of the medical curriculum at the College of Medicine King Saud bin Abdulaziz University for Health Sciences, Riyadh, Saudi Arabia

**Session number**	**Topic**
1	Evidence-Based Medicine
2	Cross Sectional Studies
3	Case-Control Studies
4	Cohort Study
5	Clinical Trials
6	Measures of Association
7	Biostatistics I
8	Biostatistics II
9	Literature Searches for the Evidence I
10	Literature Searches for the Evidence II
11	Reading Health Care Literature
12	Writing Grant Proposal
13	Writing Scientific Report
14	Overview of EBM Curriculum

**Phase II** – This phase is composed of nine blocks divided into two years. The first year includes Foundation Studies, Musculoskeletal Drug and Alcohol Dependence, Respiratory Sciences, Hematology, and Cardiovascular Sciences. The second year includes Neurosciences, Vision and Behavior; Endocrine, Nutrition and Gastroenterology; Renal, Reproduction and Sexual Health; and Oncology and Palliative Care.

Teaching and learning throughout this phase are supplemented by EBM sessions, with two EBM sessions integrated in each block. The first EBM session of each block is a large-group presentation of an EBM concept. The second EBM session is a small-group session in which students practice the skills demonstrated in the first session. The small-group sessions are facilitated by tutors with skills in EBM, and students are expected to be prepared before the session. The EBM educational program during phase II is divided into two major themes: concepts and appraisal.

**Year 1** – The first year is composed of five blocks as summarized in Table 2. The first block is the “Foundation Studies” where the EBM teaching includes an introduction to EBM and PICO question (Problems or Patients, Intervention or exposure of interest, Comparison group if available, Outcomes of interest). More specifically, the importance of and need for EBM is learned in this block. Students learn the basics of a PICO question. In “Musculoskeletal Drug and Alcohol Dependence”, the second block, the EBM topics include an overview of non-experimental studies, mainly cross-sectional, cohort, and case-control studies. This is an overview of the specific study designs, as well as strengths and limitations of study design. In the third block, Respiratory Sciences, experimental designs (mainly clinical trials) are covered. Specific issues relating to clinical trials are summarized, including randomization, allocation concealment, and blinding, as well as strengths and limitations of such designs. The fourth block is Hematology; it includes the measures of effects such as the Odds Ratio (OR), Relative Risk (RR), and Risk Difference (RD) while focusing on their use and methods of calculation. Cardiovascular Science is the final block; it includes the EBM topics of systematic reviews and specifically the process of carrying out such a review, including study identification, review process, data abstraction, and the basics of meta-analyses.

**Table 2.** EBM “concepts” included in the different blocks of the first year of phase II of the medical curriculum at the College of Medicine King Saud bin Abdulaziz University for Health Sciences, Riyadh, Saudi Arabia

**Block number**	**Block name**	**Topic**
1	Foundation Studies	Introduction to EBM and PICO question
2	Musculoskeletal Drug and Alcohol Dependence	Non-experimental study designs (cross-sectional, cohort, and case-controls)
3	Respiratory	Experimental study design (clinical trials)
4	Hematology	Measures of effect (OR, RR, RD, etc)
5	Cardiovascular	Systematic reviews and meta-analyses

**Year 2** – There are 4 blocks in the second year (Table 3). In Year 2, the EBM curriculum is directed towards teaching appraisal skills in addition to the searching skills. We convene two sessions, each of two hours’ duration, in each block except the Oncology block where we convene one session only. Thus, the total number of sessions in the second year is 7. Each session is divided into 2 activities; the first is a 30 minute lecture conducted by a faculty member which is then followed by a tutorial. Both the lecture and the tutorial cover the same topic (e.g., critical appraisal of an article on therapy).

**Table 3.** EBM “appraisal” topics included in the blocks of the second year of phase II of the medical curriculum at the College of Medicine King Saud bin Abdulaziz University for Health Sciences, Riyadh, Saudi Arabia

**Block number**	**Block name**	**Topic**
1	Neurosciences, Vision and Behavior	Appraisal of diagnosis studies, and causation
2	Endocrine, Nutrition and Gastroenterology	Appraisal of therapy studies, and advanced literature search
3	Renal, Reproduction and Sexual Health	Appraisal of prognosis studies, and using guidelines in clinical practice
4	Oncology and Palliative Care	Appraisal of meta-analyses paper

Six of the 7 sessions teach the appraisal skills; each guides the medical student through a clinical scenario. Topics include diagnosis, etiology, therapy, prognosis, systematic review, and clinical practice guidelines. The faculty members identify published journal articles related to the block for appraisal (e.g., an oncology paper will be selected for appraisal during the oncology block) and that are also related to the questions raised (e.g., therapy). The main aim of this session is the critical appraisal of the topic, not the content of the paper. A clinical scenario and the educational objectives are developed for each journal article by the faculty members.

The students are required to read the article under consideration at least one week before the session, and are also asked to read the corresponding chapter from the “Doctor's guide to EBM” textbook^4^ or other resources. The session starts with the definition of objectives, reviewing the clinical scenario, building up the clinical question (PICO), and summarizing the article (the methods and results). Then students start to appraise the article, guided by the faculty member, using the worksheets available in the “EBM Working Group's Users' Guides to the Medical Literature.”^5^

The seventh session is a practical session on searching for the evidence using computer-based training. In this session, the students choose a clinical question and search for the evidence using PubMed, ACP Journal Club, Cochrane Library and Clinical Evidence.

Student assessment is carried out in the form of Multiple Choice Questions (MCQ) incorporated in each block exam (mid-block and end-of-block) based on the exam blueprint. This method of student assessment has been approved by the Curriculum Committee. Out of 40 MCQs representing any mid block exam of a given block, 2 are allocated for the EBM topic covered during the block, whereas 4 out of the 80 MCQs representing EBM topics are included in the end of block exam. Moreover, we are in the process of improving the assessment to include continuous evaluation based on students’ participation during the sessions.

Evaluation of the whole process related to EBM is done through multiple modalities. The first and most important evaluation of the EBM curriculum is carried out by the EBM working group, which meets on regular basis. Modification of the EBM curriculum takes place as a result of the ongoing evaluation process carried out by that group. The Curriculum Committee also evaluates the EBM curriculum. Further evaluation of the program depends on block evaluation (carried out at the end of each block by the Evaluation Committee), Block coordinators’ evaluation (carried out by the students), and students’ feedback (provided by the students at the end of the block).

**Example:** The following is an example of integration of EBM in the Cardiovascular block. Two sessions are incorporated within the 7-week block, covering meta-analyses and systematic reviews.

In session 1, the following clinical scenario is presented and discussed with the students:

Sahar Ahmad, a 48-year-old teacher, presented to the family medicine clinic with high blood pressure (165/95 mm/ Hg). It was the 3^rd^ consecutive high reading within 1 month. You confirmed Sahar has hypertension and advised her to take low salt diet and do physical exercise. You ordered EKG, CXR, renal function test and lipid profile as well. Two months later her blood pressure was 155/90 mm of Hg. The investigations were normal except for high lipids. You prescribed Metoprolol (50 mg) and Statin (20 mg) daily. One of your colleagues stated that β-blockers are not considered as the first-line drug based on recent evidence. You were not aware of this information and decided to find out the latest evidence.

At that point, a discussion with the tutor is carried out about possible questions and the students are asked to formulate a focused answerable clinical question (PICO). This is followed by addressing the best evidence to look for and the potential search strategies to follow. The tutor then delivers a short presentation about the process of carrying out a systematic review, as well as meta-analysis, and further discuses potential challenges and biases of such a study design. The students are then provided with the following article: Re-examining the efficacy of β-blockers for the treatment of hypertension: a meta-analysis.^6^

In session 2, the article and worksheet for appraising an overview are distributed 3 days prior to the session. The students are given 15 minutes to organize their answers, followed by a group discussion about the validity, results, and applicability of the article. Finally, the session is concluded by reaching the proper solution to the problem.

## Discussion

Physicians and other health care providers need to understand and implement the EBM principles to improve patient care. Interactive and integrated clinical teaching and learning activities provide the basis for the best educational practice.^7^ Teaching EBM requires an educational paradigm shift, as students need to possess additional skills that are not usually part of medical training. These include the ability to answer questions rather than just “knowing the answer to questions.”^8^ This ability is critical to the development of life-long learners, an important objective of a PBL curriculum.

An EBM curriculum should foster critical thinking whereby students learn to frame options, analyze data, and understand the uncertainties that remain. We implemented faculty development programs to train the trainer, made necessary curriculum changes, and formed an EBM interest group. All these have led to rapid acceptance of EBM in our curriculum.^9^

We encourage our students to make EBM practice a *culture,* not just a course to pass. We train our students to use DynaMed,^10^ the Cochrane Library,^11^ and UpToDate,^12^ as these databases are updated frequently and contain references to the best available evidence in medical practice.

Integrating EBM in our medical curriculum was met by a few challenges, some educational and others administrative. Students admitted to our College of Medicine come from different universities with an undergraduate degree in science where EBM is not taught. Thus, introducing EBM at the early stages of the medical school as well as highlighting the importance of EBM in a physician's career is a real educational challenge. Adoption of the University of Sydney's curriculum also posed an educational dilemma for us as the University of Sydney's curriculum already had EBM integrated, but it was felt to be insufficient. We had to tailor the EBM topics over the first two years in a sequence to help students build up their expertise in EBM. We also faced some administrative challenges in the integration process. The Curriculum Committee at the College of Medicine approves any change in the curriculum. Thus, any proposed change needed to go through the Curriculum Committee.

In conclusion, we defined four basic competencies to be gained in EBM: recognition of a patient problem and construction of a structured clinical question; ability to efficiently and effectively search the medical literature to retrieve the best available evidence to answer the clinical question; critical appraisal of the evidence; and integration of the evidence with all aspects of individual patient decision making to determine the best clinical care for the patient. With the current EBM curriculum we aim to graduate self-directed, problem-based, and up-to-date physicians.

## References

[1] Sackett DL, Rosenberg WM, Gray JA, Haynes RB, Richardson WS (1996). Evidence based medicine: what it is and what it isn't. BMJ.

[2] Elstein AS (2004). On the origins and development of evidence-based medicine and medical decision making. Inflamm Res.

[3] Carlile S, Barnet S, Sefton A, Uther J (1998). Medical problem based learning supported by intranet technology: a natural student centred approach. Int J Med Inform.

[4] Hajeer AH, AlKnawy BA (2006). Doctor's Guide to Evidence-Based Medicine.

[5] Guyatt GH, Haynes RB, Jaeschke RZ, Cook DJ, Green L, Naylor CD (2000). Users' Guides to the Medical Literature: XXV. Evidence-based medicine: principles for applying the Users' Guides to patient care. Evidence-Based Medicine Working Group. JAMA.

[6] Khan N, McAlister FA (2006). Re-examining the efficacy of beta-blockers for the treatment of hypertension: a meta-analysis. CMAJ.

[7] Khan KS, Coomarasamy A (2006). A hierarchy of effective teaching and learning to acquire competence in evidenced-based medicine. BMC Med Educ.

[8] Atiya AS (2002). Teaching of evidence-based medicine to medical undergraduates. Med J Malaysia.

[9] Riegelman RK, Garr DR (2008). Evidence-based public health education as preparation for medical school. Acad Med.

[10] Alper BS (2003). DynaMed is evidence based. Fam Med.

[11] Clarke M (2007). The Cochrane Collaboration and the Cochrane Library. Otolaryngol Head Neck Surg.

[12] Fox GN, Moawad N (2003). UpToDate: a comprehensive clinical database. J Fam Pract..

